# Safe Administration of Octenidine in a Surgical Patient With Documented Severe Chlorhexidine Anaphylaxis: A Case Report and Review of the Current Evidence

**DOI:** 10.1155/cria/5754614

**Published:** 2025-09-29

**Authors:** Mathias Froidevaux, Sophie Vandenberghe-Dürr, Amélie Borgeat, Julien Maillard

**Affiliations:** ^1^ Department of Acute Care Medicine, Division of Anesthesiology, Geneva University Hospitals, Geneva, Switzerland, hug-ge.ch; ^2^ Department of Medicine, Immunology and Allergology Division, Geneva University Hospitals, Geneva, Switzerland, hug-ge.ch; ^3^ Department of Medicine, Immunology and Allergology Division, Valais Hospital, Sion, Valais, Switzerland

**Keywords:** allergy, anesthesia, chlorhexidine, cross-reactivity, octenidine, surgery

## Abstract

Chlorhexidine is one of the most widely used antiseptics worldwide. Owing to its extensive use, reports of chlorhexidine allergic reactions are increasing. In cases of severe chlorhexidine anaphylaxis, the potential for cross‐reactivity with other antiseptics poses a significant challenge when considering an alternative. We present a case of successful use of octenidine in a patient with confirmed chlorhexidine anaphylaxis, illustrating the low risk of systematic cross‐sensitivity, as already suggested by ex vivo studies, and raising the possibility of its potential use as a reliable alternative.

**Summary**

• While in vitro cross‐reactivity between chlorhexidine and octenidine has been demonstrated, its in vivo relevance remains uncertain.
• Octenidine appears to be associated with a low risk of anaphylaxis.
• Further studies are required to better define the safety profile of octenidine in patients with a documented history of severe chlorhexidine‐induced anaphylaxis.

## 1. Background/Introduction

Chlorhexidine is a widely used antiseptic with broad‐spectrum action. It is extensively employed in healthcare settings, including procedures inside and outside the operating room, from intravenous access to coronary angiography, as well as in everyday home care products such as toothpaste and cosmetics. To date, despite an increasing number of reports in the literature [1], chlorhexidine anaphylaxis is still considered rare and is likely underdiagnosed.

Systemic hypersensitivity reactions including anaphylaxis represent a rare but serious complication of general anesthesia, with an estimated incidence of approximately 1 in 10,000 procedures. Chlorhexidine accounts for up to 9% of identified culprits, ranking just behind neuromuscular blocking agents and antibiotics [2]. The allergenic potential of chlorhexidine has been attributed to its divalent biguanide or chlorophenyl groups; however, patients may also develop specific Immunoglobulin E (sIgE) directed against its hexamethylene structure, a feature shared by certain other antiseptics [3]. In light of these considerations and given the widespread use of chlorhexidine in healthcare, systematic testing for chlorhexidine allergy should be considered in all patients with allergic reactions in a healthcare setting.

Octenidine is another antiseptic used in European countries including Switzerland [4]. The molecule shares similar hexamethylene groups with chlorhexidine, raising concerns about potential cross‐reactivity. Although this phenomenon has been observed *in* vitro, evidence of its clinical relevance in vivo remains scarce.

In this case report, we describe a patient with a history of severe chlorhexidine‐induced anaphylaxis who subsequently demonstrated good tolerance to octenidine. The case report was prepared in accordance with the CARE guidelines.

## 2. Case Presentation

A 60‐year‐old male patient was scheduled to undergo an elective open distal splenopancreatectomy due to pancreatic adenocarcinoma. His medical history was notable for active smoking, hiatal hernia, and previous lumbar disc surgery. He reported no known allergies or other significant comorbidities. Both the physical examination and laboratory investigations were within normal limits.

The anesthetic plan consisted of general anesthesia with orotracheal intubation, performed as a rapid sequence induction owing to the presence of a symptomatic hiatal hernia. Analgesia was provided via an epidural catheter. In addition, radial arterial and urinary catheterization were planned for intraoperative monitoring and management. A peripheral intravenous catheter was first inserted in the patient’s arm. Midazolam was administered for anxiolysis, followed by lidocaine for local anesthesia prior to epidural catheter placement. The catheter position was verified using lidocaine with epinephrine. Sufentanil was then administered, and local anesthesia of the vein was achieved with lidocaine before induction with propofol. Orotracheal intubation was performed uneventfully by direct laryngoscopy after neuromuscular blockade with succinylcholine, which was subsequently maintained with atracurium. Dexamethasone was administered for prophylaxis of postoperative nausea and vomiting. Radial arterial and urinary catheters were inserted after induction. Skin and mucosal disinfection was performed exclusively with chlorhexidine solutions. At this stage, no antibiotic had been given, and the surgeon had not yet initiated skin preparation. A few minutes later, the patient developed refractory hypotension and bradycardia without respiratory compromise. A diluted intravenous bolus of epinephrine (50 mcg) combined with crystalloid resuscitation was administered, resulting in stabilization of blood pressure within minutes. Subsequently, the patient developed tachycardia associated with a diffuse rash. Continuous epinephrine infusion (0.04 mcg/kg/h) was initiated and supplemented with corticosteroids and an H1 antihistamine as adjuvant therapy.

The surgical procedure was postponed, and the patient was transferred to the intensive care unit. He required continuous epinephrine infusion for 2 hours, followed by an additional 4 hours of norepinephrine infusion. After a total of 6 hours, his condition had improved significantly, allowing for extubation without further complications. Thirty minutes after the reaction, tryptase levels were 18.8 mcg/L (reference value < 11 mcg/L) and returned to normal at 4.3 mcg/L the following day, confirming the anaphylactic nature of the event.

### 2.1. Investigations and Subsequent Surgery

Surgery was rescheduled 3 weeks later, as the oncologic urgency precluded the recommended 4–6 weeks interval required for comprehensive allergy testing [5]. Review of the previous anesthetic management, together with consideration of the usual incidence of perioperative anaphylaxis, identified suxamethonium, rocuronium, chlorhexidine, and latex as the most plausible trigger [2, 6].

During the second surgical procedure, both povidone iodine and octenidine were considered as alternative antiseptics. In light of the unidentified allergen and the prevailing belief that povidone hypersensitivity is more common, octenidine was selected under the erroneous assumption of a safer pharmacological profile. The patient exhibited no signs of hypersensitivity, and the surgery proceeded uneventfully. Propofol and midazolam were readministered, along with fentanyl and atracurium, all of which were well‐tolerated.

### 2.2. Allergologic Follow‐Up

A comprehensive allergy workup, based on the 2019 European Academy of Allergy and Clinical Immunology position paper, was performed two months after surgery [6] (Table 1). Skin tests were negative for suxamethonium, rocuronium, latex, and lidocaine, as were allergen‐sIgE assays for suxamethonium, quaternary ammonium, and pholcodine. Both skin and sIgE testing for chlorhexidine were positive. As midazolam had been well‐tolerated during the second intervention, it was not further tested. During the second surgical procedure, re‐exposure to lidocaine, rocuronium, succinylcholine, and propofol occurred without incident. It is also likely that the patient was re‐exposed to latex.

**Table 1:** Full allergic test workup.

Substance	Concentration	Prick test	Intradermal test	sIgE
Latex	NT	NT	NT	Negative
Chlorhexidine	5 mg/mL	+ (6 mm)	NT	1.29 kU/L
Rocuronium	10 mg/mL	1/1: −	10^E^ − 4/10^E^ − 2: −	NT
Succinylcholine	20 mg/mL	1/1: −	10^E^ − 4/10^E^ − 2: −	Negative
Lidocaine	100 mg/mL	1/1: −	10^E^ − 2/10^E^ − 1: −	NT

Several months later, the patient was accidentally re‐exposed to chlorhexidine during a peripheral venous puncture. This subsequent exposure resulted in a likely anaphylactic reaction, characterized by hypotension and tachycardia. Prompt administration of adrenaline and antihistamines was required to stabilize the patient, thereby substantiating the diagnosis of chlorhexidine hypersensitivity. However, tryptase levels were not measured during this episode.

## 3. Discussion

To the best of our knowledge, this report constitutes the first documented case of successful tolerance to octenidine in a patient with a history of severe chlorhexidine‐induced anaphylaxis in the perioperative setting.

Following perioperative anaphylaxis, a causal agent is identified in only 50%–60% of cases with skin or in vitro tests. To reduce the risk of false negatives, an allergic workup is generally recommended 4–6 weeks after the initial reaction [5]. In the present case, measurement of sIgE could have been considered before surgery, as, unlike skin tests, its diagnostic sensitivity does not appear to be significantly reduced in the immediate postanaphylaxis period [7].

Chlorhexidine‐induced anaphylaxis, while still rare (incidence 0.78 per 100,000 exposures), accounts for 5%–10% of perioperative anaphylactic reactions [2]. Over the last decade, the prevalence of chlorhexidine‐related anaphylaxis has increased. Chlorhexidine is often referred to as a “hidden allergen,” because its presence in a wide range of products, such as alcohol‐based hand gels, lubricating gels, coated central venous catheters, disinfectant swabs and wound cleansers, is frequently overlooked and is usually not mentioned in anesthesia reports. The rising sensitization rate, particularly among healthcare professionals, may be explained by its extensive use in items including hand sanitizers, wound disinfectants, toothpaste, mouthwashes, contact lens solutions, and cosmetics. In addition, improved standardization of allergy testing has likely contributed to the greater recognition of this condition [6].

Octenidine is an antiseptic with activity against both Gram‐negative and Gram‐positive organisms, predominantly used in European countries since the 1980s. Developed in Germany, it has been authorized by the European Medicines Agency (EMA) but not by the U.S. Food and Drug Administration (FDA). Evidence regarding its clinical effectiveness in preventing surgical site infections remains limited, as only a few studies have directly assessed its efficacy relative to other antiseptics [4]. Octenidine and chlorhexidine share a similar structural hexamethylene group, and cross‐reactivity of IgE antibodies between the two compounds has been demonstrated in vitro [3, 8].

In routine clinical practice, octenidine has demonstrated a favorable safety profile, with only a few cases of anaphylaxis reported since its introduction in the late 1980s [9, 10]. However, the clinical significance of in vitro findings remains uncertain. Boag et al. described a case of severe anaphylaxis attributed to octenidine, despite negative results on both skin prick testing and topical challenge; notably, chlorhexidine was not included in the testing protocol [10]. Mueller‐Wirth et al. reported another case of severe octenidine‐induced anaphylaxis, in which both skin tests and basophil activation tests were positive [9]. Additional assays revealed sensitization to hexamethylene‐containing compounds (chlorhexidine, alexidine, and polyhexanide), suggesting that this structural compound may represent a shared IgE epitope responsible for cross‐reactivity among antiseptics. Shunter et al. also described a case of life‐threatening anaphylaxis involving cross‐reactivity between chlorhexidine and polyhexanide, an antiseptic containing both hexamethylene and biguanide groups, while testing for octenidine remained negative [11]. In our patient, despite severe anaphylaxis to chlorhexidine, no clinical evidence of cross‐reactivity with octenidine was observed.

**Figure 1:**
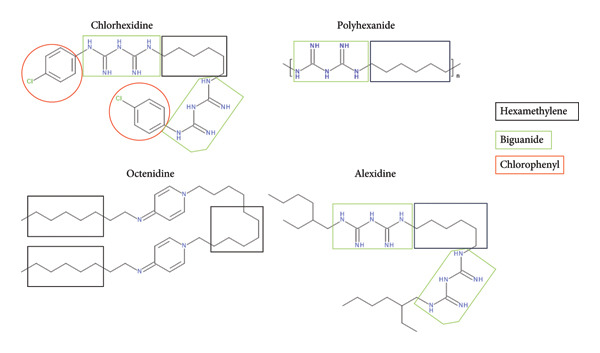
Molecular structures of common disinfectants.

The allergenic potential of chlorhexidine is most likely related to the presence of its divalent biguanide or chlorophenyl groups [9]. In addition, patients may develop specific IgE antibodies directed against the monovalent hexamethylene moiety (Figure 1). While the chlorophenyl group is unique to chlorhexidine, the hexamethylene moiety is also found in alexidine, polyhexanide, and octenidine. Similarly, the biguanide groups are shared by alexidine and polyhexanide.

Mueller‐Wirth et al. analyzed the sera of 44 patients with confirmed chlorhexidine allergy to assess potential cross‐reactivity toward the other disinfectants alexidine, polyhexanide, and octenidine, using skin and basophil activation tests as well as inhibition assays [3]. Their findings suggest that the IgE response elicited by chlorhexidine sensitization is likely polyclonal. This interpretation is supported by the observation that the sera from 33% of chlorhexidine allergic patients displayed cross‐reactivity with other disinfectants. Notably, only one patient had experienced anaphylaxis to chlorhexidine with potential clinical cross‐reactivity to octenidine (contact eczema) and multiple cross‐reactive in vitro tests, suggesting sensitization to the common hexamethylene structure. A smaller case series by Ebo et al. employed passive mast cell activation tests to explore cross‐sensitivity between chlorhexidine and other disinfectants [8]. This study demonstrated that 30% of chlorhexidine allergic patients exhibited in vitro cross‐reactivity to octenidine at high concentrations; however, the clinical significance of this finding remains uncertain.

In our patient, it can be hypothesized that sensitization occurred primarily against the biguanide and/or chlorophenyl moieties, while no IgE response developed to the hexamethylene groups. The absence of such sensitization likely explains the lack of cross‐reactivity to octenidine (Figure 1).

At present, sIgE testing for chlorhexidine remains the only routinely available laboratory assay, thereby limiting the evaluation of potential cross‐sensitization. Moreover, the lack of standardized skin test protocols for alternative disinfectants increases the risk of false‐positive (irritative) reactions. In this context, in vitro cellular assays, such as the basophil activation test, may provide valuable complementary information.

Further studies are warranted to clarify both the frequency and clinical significance of cross‐reactivity among disinfectants sharing allergenic determinants, such as hexamethylene or biguanide groups. A better understanding of these mechanisms would provide valuable guidance for clinicians managing patients at risk of severe, potentially life‐threatening anaphylaxis.

## Consent

The patient has given written informed consent for this publication.

## Conflicts of Interest

The authors declare no conflicts of interest.

## Funding

No funding was received for this study.
